# Determinants of outcomes following surgery for type A acute aortic dissection: the UK National Adult Cardiac Surgical Audit

**DOI:** 10.1093/eurheartj/ehab586

**Published:** 2021-09-01

**Authors:** Umberto Benedetto, Arnaldo Dimagli, Amit Kaura, Shubhra Sinha, Giovanni Mariscalco, George Krasopoulos, Narain Moorjani, Mark Field, Trivedi Uday, Simon Kendal, Graham Cooper, Rakesh Uppal, Haris Bilal, Jorge Mascaro, Andrew Goodwin, Gianni Angelini, Geoffry Tsang, Enoch Akowuah

**Affiliations:** Bristol Heart Institute, University of Bristol, Senate House, Tyndall Avenue, Bristol BS8 1TH, UK; Bristol Heart Institute, University of Bristol, Senate House, Tyndall Avenue, Bristol BS8 1TH, UK; National Institute for Health Research Imperial Biomedical Research Centre, Imperial College London and Imperial College Healthcare NHS Trust, The Bays, South Wharf Road, St Mary's Hospital, London W21NY, UK; Bristol Heart Institute, University of Bristol, Senate House, Tyndall Avenue, Bristol BS8 1TH, UK; Department of Cardiac Surgery, Glenfield Hospital, Groby Rd, Leicester LE3 9QP, UK; Oxford University Hospitals NHS Foundation Trust, Headley Way, Headington, Oxford OX3 9DU, UK; Department of Cardiothoracic Surgery, Royal Papworth Hospital, Papworth Rd, Trumpington, Cambridge CB2 0AY, UK; Department of Cardiothoracic Surgery, Liverpool Heart and Chest Hospital, Thomas Dr, Liverpool L14 3PE, UK; Sussex Cardiac Center, Brighton and Sussex University Hospitals NHS Trust, Barry Building, Eastern Rd, Brighton BN2 5BE, UK; South Tees Hospitals NHS Trust, Marton Road, Middlesbrough TS4 3BW, UK; Sheffield Teaching Hospitals Foundation Trust, Royal Hallamshire Hospital, Glossop Rd, Broomhall, Sheffield S10 2JF, UK; Barts Heart Centre, William Harvey Research Institute, W Smithfield, London EC1A 7BE, UK; Department of Cardiothoracic Surgery, Manchester Royal Infirmary, Oxford Rd, Manchester M13 9WL, UK; University Hospital Birmingham NHS Foundation Trust, Mindelsohn Way, Edgbaston, Birmingham, B15 2GW, UK; South Tees Hospitals NHS Trust, Marton Road, Middlesbrough TS4 3BW, UK; Bristol Heart Institute, University of Bristol, Senate House, Tyndall Avenue, Bristol BS8 1TH, UK; Wessex Cardiothoracic Center, University Hospital Southampton NHS Trust, Tremona Road Southampton, Hampshire SO16 6YD, UK; South Tees Hospitals NHS Trust, Marton Road, Middlesbrough TS4 3BW, UK

**Keywords:** Type A aortic dissection, Risk score, Volume–outcome relationship, Risk factors

## Abstract

**Aims:**

Operability of type A acute aortic dissections (TAAAD) is currently based on non-standardized decision-making process, and it lacks a disease-specific risk evaluation model that can predict mortality. We investigated patient, intraoperative data, surgeon, and centre-related variables for patients who underwent TAAAD in the UK.

**Methods and results:**

We identified 4203 patients undergoing TAAAD surgery in the UK (2009–18), who were enrolled into the UK National Adult Cardiac Surgical Audit dataset. The primary outcome was operative mortality. A multivariable logistic regression analysis was performed with fast backward elimination of variables and the bootstrap-based optimism-correction was adopted to assess model performance. Variation related to hospital or surgeon effects were quantified by a generalized mixed linear model and risk-adjusted funnel plots by displaying the individual standardized mortality ratio against expected deaths. Final variables retained in the model were: age [odds ratio (OR) 1.02, 95% confidence interval (CI) 1.02–1.03; *P* < 0.001]; malperfusion (OR 1.79, 95% CI 1.51–2.12; *P* < 0.001); left ventricular ejection fraction (moderate: OR 1.40, 95% CI 1.14–1.71; *P* = 0.001; poor: OR 2.83, 95% CI 1.90–4.21; *P* < 0.001); previous cardiac surgery (OR 2.29, 95% CI 1.71–3.07; *P* < 0.001); preoperative mechanical ventilation (OR 2.76, 95% CI 2.00–3.80; *P* < 0.001); preoperative resuscitation (OR 3.36, 95% CI 1.14–9.87; *P* = 0.028); and concomitant coronary artery bypass grafting (OR 2.29, 95% CI 1.86–2.83; *P* < 0.001). We found a significant inverse relationship between surgeons but not centre annual volume with outcomes.

**Conclusions:**

Patient characteristics, intraoperative factors, cardiac centre, and high-volume surgeons are strong determinants of outcomes following TAAAD surgery. These findings may help refining clinical decision-making, supporting patient counselling and be used by policy makers for quality assurance and service provision improvement.

## Graphical Abstract

**Figure:**
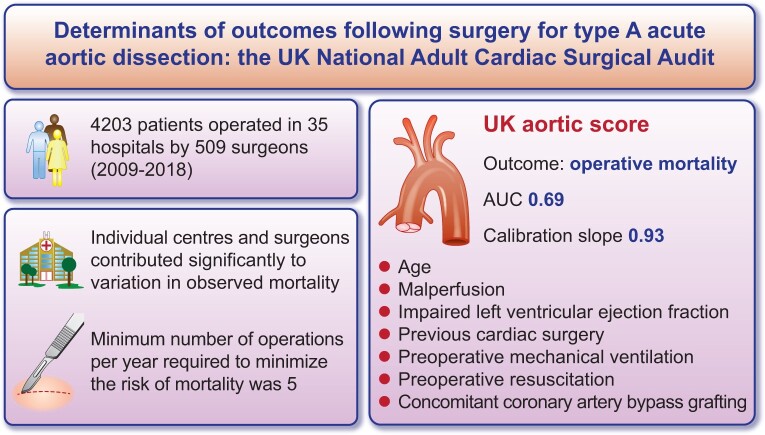

**See page 53 for the editorial comment for this article ‘Acute type A aortic dissection reconsidered: it’s all about the location of the primary entry tear and the presence or absence of malperfusion’, by M. Czerny and B. Rylski, https://doi.org/10.1093/eurheartj/ehab664.**

## Introduction

Type A acute aortic dissection (TAAAD) is a life-threatening condition associated with the significant risk of mortality and morbidity. Mortality for TAAAD is 50% by 24 h and 50% of patients die before reaching a specialist centre.^1–5^ Prompt surgical repair remains the standard treatment for these patients. Improvement in diagnostic techniques, initial management, and increased clinical awareness^6^ over the last decade are expected to have increased the number of patients promptly diagnosed and referred to surgery. However, survival after surgical repair is still suboptimal, with high in-hospital mortality (16–18%).^3^ ^,^ ^7–10^ Controversy still exists about which factors should be considered during the preoperative evaluation and decision-making process that can assess risk and predict operative mortality. Moreover, the impact of different surgical strategies on outcomes remains unclear.^10–12^ The impact of the centre or surgeon volume–outcome relationship on mortality remains poorly understood. A better understanding of outcome determinants in patients undergoing surgery could support decision-making, help when designing service provision, and improve outcomes of the surviving patients that reach specialist centres. Moreover, a precise risk stratification can provide better patient counselling and be used for unit and surgeon benchmarking. In the present study, we aimed to investigate predictors of outcome in patients undergoing surgery for TAAAD, including clinical, perioperative, centre, and surgeon-level variables.

## Methods

This study is part of a research project approved by the Health Research Authority and Health and Care Research Wales. Patient consent was waived (HCRW**)** (IRAS ID: 278171) in accordance with the research guidance. This study complies with the Declaration of Helsinki.

### Data extraction and cleaning

Complete extraction of prospectively collected data from the National Adult Cardiac Surgery Audit (NACSA) was obtained from the National Institute for Cardiovascular Outcomes Research (NICOR) central cardiac database and retrospectively analysed. The definitions of the database variables used for this study are available at https://www.nicor.org.uk/national-cardiac-audit-programme/adult-cardiac-surgery-surgery-audit/. The NICOR registry prospectively collects demographic, as well as pre- and post-operative clinical information, including mortality, for all major adult cardiac surgery procedures performed in the UK. The data flow from surgeon input to analysis has been described elsewhere.^13^ Briefly, data entered locally by surgeons are validated at the unit level by database managers prior to upload via a web portal to NICOR. At this stage, further validation is performed according to logical rules and missing data reports are generated for primary variables (e.g. EuroSCORE risk factors, patient identifiers and outcome data). The data are then forwarded to an academic healthcare informatics department for data cleaning. The complete data cleaning process has been described previously.^13^ Duplicate records are removed, transcriptional discrepancies re-coded, and clinical and temporal conflicts resolved. Missing data are resolved during the validation stages of the data transfer from individual centres. Missing and conflicting data for in-hospital mortality are backfilled and validated via record linkage to the Office for National Statistics census database*.* The overall percentage of missing data for baseline information is very low (1.7%). Missing categorical or dichotomous variable data were imputed with the mode while missing continuous variables data were imputed with the median. For the present analysis, from the NACSA registry, we identified patients undergoing surgery for TAAAD from January 2009 to December 2018 in England, Scotland, and Wales. All procedures included in the present analysis were classified under the heading of urgent (non-elective admission with need for surgery during the same admission), emergency (operation before the next working day), or salvage (patients needing cardiopulmonary resuscitation on route to theatre or before anaesthesia induction). We calculated the annual and total number of cases (volume) performed by responsible surgeon and cardiac centre.

### Outcomes

The primary outcome was in-hospital mortality. Other outcomes investigated were postoperative non-fatal cerebrovascular events, need for postoperative dialysis, and re-exploration for bleeding.

### Statistical analysis

Categorical variables were summarized as counts and percentages. Continuous variables were summarized as median (interquartile range). Patient’s characteristics, operative data, and outcomes were presented in the overall sample and stratified in three groups by the extent of surgical repair: interposition graft, total aortic root replacement, and aortic arch replacement (with or without aortic root replacement). Comparison of variable distribution between groups was performed by means of χ^2^ test or Kruskal–Wallis test. To investigate determinants of survival, a stepwise approach was adopted.

#### Analysis on risk factors

We performed a multivariate logistic regression analysis on in-hospital mortality based on patient characteristics at presentation and surgical data. Fast backward elimination on factors, using a method based on Lawless and Singhal,^14^ was implemented. This method uses the fitted complete model and computes approximate Wald statistics by computing conditional (restricted) maximum likelihood estimates assuming multivariate normality of estimates. *P*-value was used as stopping rule and a *P*-value of 0.05 was used as significance level for a variable to remain in the final model (model 1). Bootstrap-based optimism-correction for the new model was performed to allow estimation of the ‘optimism’ inherent in a predictive accuracy measure derived from model training and testing on the same sample (i.e. the *apparent* accuracy). This optimism is estimated by taking bootstrap samples from the full data, and for each sample, one carries out the same model development procedure applied to the full data and then evaluates performance of the resulting model on the bootstrap sample it was developed on, and also the full sample. The difference between these performance estimates is then averaged over bootstrap samples, to obtain an estimate of the optimism, which is then subtracted from the apparent accuracy.

We also externally validated the risk model derived from the International Registry of Aortic Dissection (IRAD).^15^ Discrimination, that is the prediction model ability to distinguish between subjects developing and not developing the outcome, was assessed using the area under the receiver operating characteristic curve (AUC). An AUC value of 0.5 indicates no discrimination ability, 0.7–0.8 indicates good discrimination ability, 0.8–0.9 excellent discrimination ability, and 0.9 outstanding performance.

#### Analysis of hospital and surgeon effect

Variation related to hospital or surgeon effect was quantified by generalized mixed linear model, which included the logit of predicted probabilities obtained from model 1, hospital and surgeon annual volume as fixed terms, and individual hospital and surgeon as random effect. ANOVA for nested model was used to investigate whether the inclusion of random effects significantly improved overall model performance. We also tested the effect of responsible anaesthetist as random effect. Non-linearity for hospital and surgeon annual volume was tested using spline term. Best cut-off points for annual volume were identified using Youden index defined as [(sensitivity + specificity) − 1]. The maximum value of the Youden index is 1 (perfect test) and the minimum is 0 when the test has no diagnostic value. Moreover, to investigate whether variation in mortality can be attributed to individual hospital or surgeon performance, risk-adjusted funnel plots were generated. Each hospital and surgeon were then displayed as a scatter point showing the individual standardized mortality ratio (risk-adjusted) against expected deaths. Standardized ratio was calculated as observed/predicted mortality rate where predicted mortality corresponded to individual risk probabilities obtained from model 1. Upper and lower control limits calculated at 3δ (corresponding to 99.8% confidence intervals) from the mean, using the exact binomial method described by Spiegelhalter.^16^ An upper warning limit (calculated similarly to 95% confidence intervals) was also calculated at 2δ from the mean. Nonparametric dispersion test via mean deviance residual fitted vs. simulated-refitted was used to assess overdispersion.

## Results

**Figure 1:**
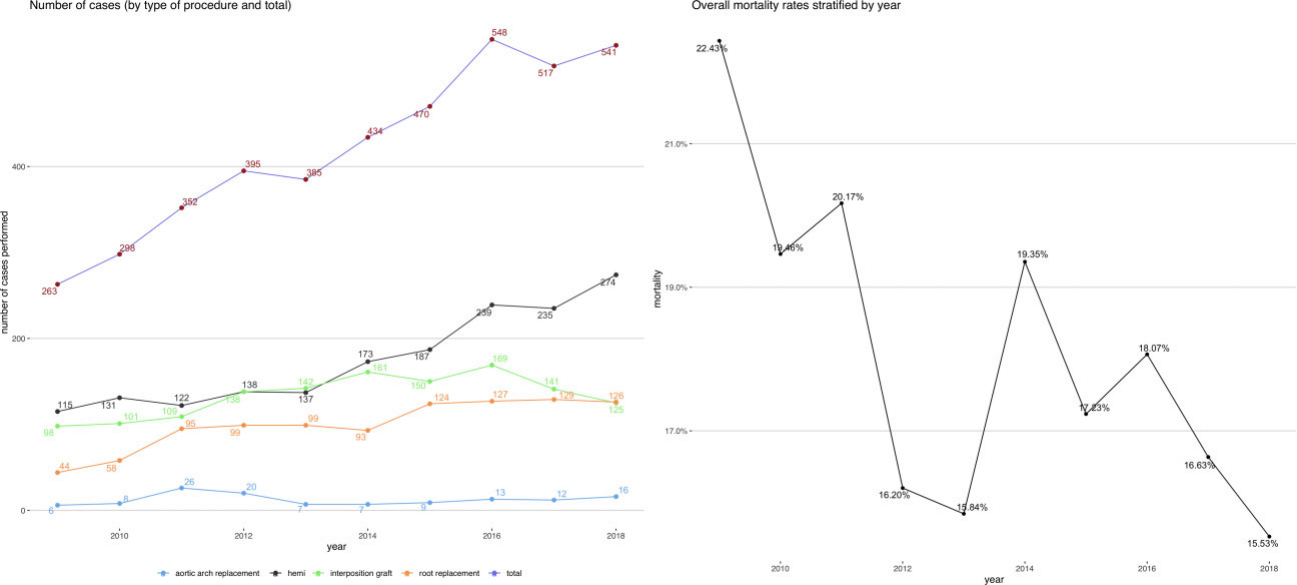
Trend in number of surgeries for type A acute aortic dissection (overall and by type of procedure) (left) and overall mortality rates (right).

A total of 4203 patients who underwent surgical repair for TAAAD in 35 hospitals by 509 surgeons from 2009 to 2018 were identified (median age 64 [52–73], 33.3% female). There was a steady increase in the number of cases performed annually over the years as shown in *Figure 1*. This corresponded to an increase in the mean number of annual cases performed by each surgeon from 2.9 to 5.8. Most patients received a hemiarch surgery (*n* = 1751, 41.7%), followed by interposition graft (*n* = 1334, 31.7%), aortic root replacement (*n* = 994, 23.6%), and only a small number of patients received total arch replacement (*n* = 124, 3.0%) and this trend was constant over time. Patient characteristics, operative data and outcomes in the overall sample and according to the extent of surgical repair are presented in *Table 1*. Patients receiving aortic root replacement tended to be younger and as expected and were more likely to have a previous diagnosis of Marfan syndrome. Patients undergoing total arch replacement were more likely to have previous cardiac surgery and were operated on in hospitals and by surgeons with a higher annual and overall volume.

**Table 1:** Patient characteristics, operative data, and outcomes in the overall sample and stratified by type of procedure

	Overall	Aortic arch replacement	Hemiarch	Interposition graft	Aortic root replacement	*P*-value
*N*	4203	124	1751	1334	994	
Eras, *n* (%)						<0.001
2009–10	561 (13.3)	14 (11.3)	246 (14.0)	199 (14.9)	102 (10.3)	
2011–12	747 (17.8)	46 (37.1)	260 (14.8)	247 (18.5)	194 (19.5)	
2013–14	819 (19.5)	14 (11.3)	310 (17.7)	303 (22.7)	192 (19.3)	
2015–16	1018 (24.2)	22 (17.7)	426 (24.3)	319 (23.9)	251 (25.3)	
2017–18	1058 (25.2)	28 (22.6)	509 (29.1)	266 (19.9)	255 (25.7)	
Age (median [IQR])	63.80 [52.30, 73.00]	62.05 [52.53, 70.15]	65.50 [55.00, 73.40]	67.60 [56.23, 75.60]	55.75 [46.40, 66.77]	<0.001
Age categories, *n* (%)						<0.001
≤59	1710 (40.7)	56 (45.2)	611 (34.9)	435 (32.6)	608 (61.2)	
60–64	506 (12.0)	19 (15.3)	238 (13.6)	134 (10.0)	115 (11.6)	
65–69	550 (13.1)	15 (12.1)	266 (15.2)	181 (13.6)	88 (8.9)	
70–74	623 (14.8)	20 (16.1)	288 (16.4)	218 (16.3)	97 (9.8)	
75–79	537 (12.8)	6 (4.8)	245 (14.0)	222 (16.6)	64 (6.4)	
≥80	277 (6.6)	8 (6.5)	103 (5.9)	144 (10.8)	22 (2.2)	
Female sex, *n* (%)	1399 (33.3)	37 (29.8)	606 (34.6)	475 (35.6)	281 (28.3)	0.001
Marfan syndrome, *n* (%)	112 (2.7)	4 (3.2)	25 (1.4)	20 (1.5)	63 (6.3)	<0.001
Chronic pulmonary disease, *n* (%)	356 (8.5)	15 (12.1)	134 (7.7)	140 (10.5)	67 (6.7)	0.002
Smoking, *n* (%)						0.003
Never smoked	2172 (51.7)	49 (39.5)	865 (49.4)	731 (54.8)	527 (53.0)	
Ex-smoker	1340 (31.9)	49 (39.5)	576 (32.9)	415 (31.1)	300 (30.2)	
Current smoker	691 (16.4)	26 (21.0)	310 (17.7)	188 (14.1)	167 (16.8)	
Hypertension, *n* (%)	2808 (66.8)	90 (72.6)	1255 (71.7)	888 (66.6)	575 (57.8)	<0.001
Diabetes, *n* (%)						0.006
Not diabetic	3968 (94.4)	117 (94.4)	1676 (95.7)	1229 (92.1)	946 (95.2)	
Diet	52 (1.2)	3 (2.4)	18 (1.0)	21 (1.6)	10 (1.0)	
Oral therapy	149 (3.5)	4 (3.2)	47 (2.7)	67 (5.0)	31 (3.1)	
Insulin	34 (0.8)	0 (0.0)	10 (0.6)	17 (1.3)	7 (0.7)	
CVA, *n* (%)	247 (5.9)	6 (4.8)	107 (6.1)	86 (6.4)	48 (4.8)	0.366
LVEF (%)						<0.001
Good (≥50%)	3349 (79.7)	101 (81.5)	1428 (81.6)	1079 (80.9)	741 (74.5)	
Moderate (30–49%)	721 (17.2)	23 (18.5)	278 (15.9)	213 (16.0)	207 (20.8)	
Poor (<30%)	133 (3.2)	0 (0.0)	45 (2.6)	42 (3.1)	46 (4.6)	
Chronic kidney disease, *n* (%)	168 (4.0)	6 (4.8)	65 (3.7)	48 (3.6)	49 (4.9)	0.333
Previous cardiac surgery, *n* (%)	254 (6.0)	16 (12.9)	92 (5.3)	73 (5.5)	73 (7.3)	0.001
Preoperative ventilation, *n* (%)	200 (4.8)	4 (3.2)	82 (4.7)	59 (4.4)	55 (5.5)	0.510
Preoperative resuscitation, *n* (%)	17 (0.4)	0 (0.0)	6 (0.3)	4 (0.3)	7 (0.7)	0.355
Ongoing chest pain, *n* (%)	450 (10.7)	16 (12.9)	197 (11.3)	135 (10.1)	102 (10.3)	0.603
Malperfusion^a^, *n* (%)	1534 (36.5)	49 (39.5)	641 (36.6)	491 (36.8)	353 (35.5)	0.809
Any pulse deficit, *n* (%)	831 (19.8)	41 (33.1)	342 (19.5)	277 (20.8)	171 (17.2)	<0.001
Acute renal failure, *n* (%)	127 (3.0)	7 (5.6)	48 (2.7)	32 (2.4)	40 (4.0)	0.036
MI at presentation, *n* (%)	124 (3.0)	1 (0.8)	42 (2.4)	31 (2.3)	50 (5.0)	<0.001
TIA in the last 24 h, *n* (%)	176 (4.2)	5 (4.0)	76 (4.3)	68 (5.1)	27 (2.7)	0.042
Cardiogenic shock, *n* (%)	609 (14.5)	6 (4.8)	259 (14.8)	193 (14.5)	151 (15.2)	0.020
Total root replacement, *n* (%)	1015 (24.1)	21 (16.9)	0 (0.0)	0 (0.0)	994 (100.0)	<0.001
Aortic root sparing, *n* (%)	22 (0.5)	2 (1.6)	0 (0.0)	0 (0.0)	20 (2.0)	<0.001
Aortic arch replacement (%)	124 (3.0)	124 (100.0)	0 (0.0)	0 (0.0)	0 (0.0)	<0.001
Hybrid endovascular, *n* (%)	68 (1.6)	68 (54.8)	0 (0.0)	0 (0.0)	0 (0.0)	<0.001
Aortic valve replacement, *n* (%)	1148 (77.8)	21 (77.8)	206 (51.2)	220 (67.5)	701 (97.2)	<0.001
Bicuspid aortic valve, *n* (%)	98 (2.3)	1 (0.8)	23 (1.3)	12 (0.9)	62 (6.2)	<0.001
Aortic regurgitation, *n* (%)	1209 (28.8)	28 (22.6)	361 (20.6)	239 (17.9)	581 (58.5)	<0.001
Cerebral perfusion, *n* (%)	1126 (26.8)	70 (56.5)	627 (35.8)	188 (14.1)	241 (24.2)	<0.001
Concomitant CABG, *n* (%)	554 (13.2)	10 (8.1)	182 (10.4)	204 (15.3)	158 (15.9)	<0.001
Circulatory arrest time, median [IQR]	25.00 [8.00, 39.00]	35.00 [16.00, 76.00]	30.00 [21.00, 44.00]	0.00 [0.00, 0.00]	22.00 [4.00, 37.00]	<0.001
Total ischaemic time, median [IQR]	110.00 [77.00, 154.00]	142.00 [92.00, 191.50]	102.00 [72.00, 135.00]	91.00 [68.00, 126.00]	153.00 [117.00, 203.00]	<0.001
Hospital annual volume, median [IQR]	16.00 [11.00, 23.00]	18.00 [13.00, 24.25]	15.00 [10.00, 22.00]	17.00 [11.00, 28.00]	15.00 [10.00, 21.00]	<0.001
Surgeon annual volume, median [IQR]	4.00 [2.00, 6.00]	5.00 [3.00, 8.00]	4.00 [2.00, 6.00]	4.00 [2.00, 6.00]	4.00 [2.00, 6.00]	<0.001
Operative mortality, *n* (%)	747 (17.8)	28 (22.6)	324 (18.5)	220 (16.5)	175 (17.6)	0.249
Non-fatal CVA, *n* (%)	388 (9.2)	18 (14.5)	195 (11.1)	103 (7.7)	72 (7.2)	<0.001
Postoperative dialysis, *n* (%)	560 (14.7)	24 (21.1)	256 (16.3)	126 (10.5)	154 (16.7)	<0.001
SWI, *n* (%)	33 (1.4)	1 (1.4)	15 (1.6)	9 (1.2)	8 (1.3)	0.917
Re-exploration, *n* (%)	441 (11.9)	18 (18.0)	180 (11.8)	100 (8.3)	143 (16.2)	<0.001

CABG, coronary artery bypass grafting; COPD, chronic obstructive pulmonary disease; CVA, cerebrovascular accident; IQR, interquartile range; LVEF, left ventricular ejection fraction; MI, myocardial infarction; SWI, sternal wound infection; TIA, transient ischaemic attack.

a Defined as one of the following conditions: coronary malperfusion, renal malperfusion, cardiogenic shock (cardiac tamponade, low cardiac output), cerebral perfusion (CVA in the previous 24 h), and any pulse deficit/limb ischaemia.

The crude mortality rate was 17.8% with evidence of a steady decrease over the years (from 22.4% in 2009 to 15.5% in 2018) (*Table 1* and *Figure 1*). Nine percentage of patients experienced non-fatal stroke, and re-exploration and postoperative dialysis were required in 11.9% and 14.7% of patients, respectively. Patients undergoing total arch replacement showed a trend towards an increased crude incidence of mortality (22.6%), non-fatal stroke (14.5%), need for dialysis (21.1%), and re-exploration (18%). There were no differences in outcomes in patients undergoing total arch replacement or hemiarch surgery with or without cerebral perfusion (Supplementary material online, *Tables S1 and S2*). Similarly, no differences in outcomes were recorded when endovascular procedure was performed in adjunct to total arch surgery (Supplementary material online, *Table S3*).

### Risk modelling for mortality and external validation of the International Registry of Aortic Dissection score

Distribution of baseline and operative data in survivors and non-survivors is presented in Supplementary material online, *Table S4*. Final variables retained in the model (*Table 2*) were: age (OR 1.02, 95% CI 1.02–1.03; *P* < 0.001), malperfusion (OR 1.79, 95% CI 1.51–2.12; *P* < 0.001), left ventricular ejection fraction (moderate: OR 1.40, 95% CI 1.14–1.71; *P* = 0.001; poor: OR 2.83, 95% CI 1.90–4.21; *P* < 0.001), previous cardiac surgery (OR 2.29, 95% CI 1.71–3.07; *P* < 0.001), preoperative mechanical ventilation (OR 2.76, 95% CI 2.00–3.80; *P* < 0.001), preoperative resuscitation (OR 3.36, 95% CI 1.14–9.87; *P* = 0.028), and concomitant coronary artery bypass grafting (OR 2.29, 95% CI 1.86–2.83; *P* < 0.001). Bootstrapping validation showed a good performance of the model (optimism corrected AUC 0.69; slope 0.93) (Supplementary material online, *Figure S1*; Supplementary material online, *Table S5*). The new risk scoring system is available online (https://cardiacsurgery.shinyapps.io/dynnomapp/).

**Table 2:** Independent risk factors included in the UK aortic score

Characteristic	OR	95% CI	*P*-value
Age	1.02	1.02–1.03	<0.001
Malperfusion^a^	1.79	1.51–2.12	<0.001
LVEF			
Good (≥50%)	–	–	
Moderate (30–49%)	1.40	1.14–1.71	0.001
Poor (<30%)	2.83	1.89–4.20	<0.001
Previous cardiac surgery	2.29	1.70–3.06	<0.001
Preoperative ventilation	2.76	1.99–3.79	<0.001
Preoperative resuscitation	3.36	1.16–10.4	0.028
CABG	2.29	1.85–2.82	<0.001

CABG, coronary artery bypass grafting; CI, confidence interval; LVEF, left ventricular ejection fraction; OR, odds ratio.

a Defined as one of the following conditions: coronary malperfusion, renal malperfusion, cardiogenic shock (cardiac tamponade, low cardiac output), cerebral perfusion (cerebrovascular accident in the previous 24 h), and any pulse deficit/limb ischaemia.

In the external validation of the IRAD score, an AUC of 0.63 and calibration slope of 0.56 were achieved in our dataset (Supplementary material online, *Figure S2*).

### Hospital and surgeon effect

The generalized mixed model showed that both individual centres (δ = 0.13, SD = 0.36; ANOVA *P* < 0.001 vs. fixed terms only) and individual surgeons (δ = 0.21, SD = 0.46; ANOVA *P* < 0.001 vs. fixed terms and hospital random effect) contributed significantly to variation in observed mortality, after controlling for patient predicted risk and annual hospital and surgeon volume. Contrarily, responsible anaesthetist was not found as source of variation (δ = 0, SD = 0; ANOVA *P* = 1 vs. fixed terms and hospital and surgeon random effect) (Supplementary material online, *Table S6*). We found evidence that surgeon (OR 0.95, 95% CI 0.92–0.99; *P* = 0.02) but not hospital volume (OR 0.99, 95% CI 0.98–1.01; *P* = 0.37) was inversely correlated with the risk of mortality (Supplementary material online, *Table S6*). Patient characteristics, operative data, and outcomes stratified by surgeon annual volume terciles are presented in Supplementary material online, *Table S7*. Mortality outcome was improved when the individual caseload was 5 cases per surgeon per year (Youden index 0.11). These surgeons (>5/year) were more likely to operate on patients who were older and had previous cardiac surgery but less likely to be in critical condition (cardiogenic shock, myocardial infarction at presentation). They were also more likely to perform aortic arch surgery and concomitant endovascular procedures and to use cerebral perfusion.

**Figure 2:**
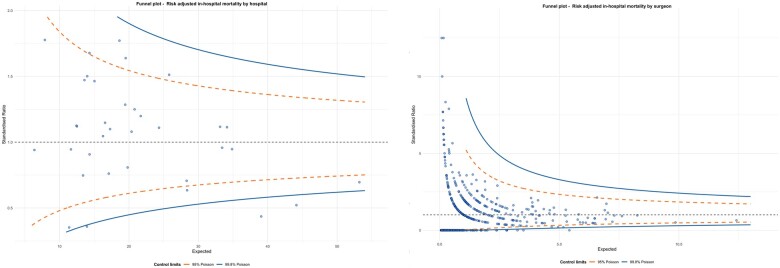
Funnel plots for hospital mortality 2009–19 in the UK stratified by hospitals (*n* = 35) (left) and surgeons (*n* = 509) (right).

Out of the 35 centres, the funnel plot shows 10 and 3 outlier hospitals outside the 95% and 99.8% alarm line (*Figure 2*, left panel). Out of 509 surgeons, 217 and 196 were found to stand outside 95% and 99.8% alarm lines, respectively (*Figure 2*, right panel). No evidence of overdispersion was found for hospital outliers (*P* = 0.33).

## Discussion

To the best of our knowledge, this is the most comprehensive analysis on determinants of outcomes after surgical repair for TAAAD. It is based on a decade (2009–18) of surgery and data collection into a recognized nationwide dataset. Our analysis focused on patient characteristics, surgical strategies, and hospital-, surgeon-, and anaesthetist-level effect including annual case volume. We found an increase in the number of total procedures and annual number of procedures performed per surgeon. Most cases were treated with hemiarch or limited replacement of the ascending aorta (interposition graft), this was followed by replacement of the aortic root and only a small proportion of patients undergoing total aortic arch replacement. The overall raw mortality rate was 18% with a decrease over time from 22% in 2009 to 15% in 2018. We identified seven independent risk factors associated with mortality: age, malperfusion, previous heart surgery, reduced left ventricular systolic function, preoperative ventilation, preoperative resuscitation, and concomitant coronary artery bypass graft surgery, which were used to build a bedside risk scoring tool, the UK aortic score (*Graphical Abstract*). This score was developed on the largest series of TAAAD surgical repairs ever reported.^15^ ^,^ ^17^ ^,^ ^18^

We also investigated whether there was any evidence of cardiac surgery centre and operating surgeon volume–outcome relationship and whether the effect on mortality has a volume–outcome relationship. We found that individual centre and surgeon showed an effect on mortality, which was independent of volume, with a larger effect related to individual surgeon and no effect related to the responsible anaesthetist. Only operating surgeon volume was associated with a lower risk of mortality, with 5 being the minimum number of TAAAD repairs per surgeon per year required to minimize the risk of mortality.

The results reported here are consistent with those reported in other national and international registries. The IRAD registry collected information on 2952 patients with type A aortic dissection from 28 referral centres from North America, Europe, Asia, and Australia and reported an operative mortality of 18%.^3^ The German Registry for Acute Aortic Dissection Type A (GERAADA) collected information on 2137 patients undergoing surgery for TAAAD in 50 centres in Germany, Austria, Switzerland, and Luxembourg from July 2006 to June 2010^7^ and reported an overall mortality of 17%. Similar mortality rates (16%) were reported by the Nordic Consortium for Acute Type A Aortic Dissection (NORCAAD), a collaborative registry of eight academic cardiothoracic centres in Denmark, Finland, Iceland, and Sweden.^8^ A retrospective analysis of 6387 patients from the Society of Thoracic Surgeons (STS) database (2014–18) reported an overall mortality of 16.5%.^9^ Notably, the mean age was similar across these registries (60–62 years) and the risk factors included in our model were consistent with those reported in other registries including co-existing comorbidities (e.g. advanced age) and critical conditions at presentation (e.g. mechanical ventilation, malperfusion).^7^ ^,^ ^15^ ^,^ ^17^ ^,^ ^19^ The definition of malperfusion is variable across the different registries. In our study, we classified malperfusion as the resultant of patients presenting with coronary malperfusion, renal malperfusion, cardiogenic shock (cardiac tamponade, low cardiac output), cerebral perfusion (cerebrovascular accidents in the previous 24 h), and any pulse deficit/limb ischaemia. The reported rate was similar to that reported in the GERAADA database^7^ and higher than that reported in the NORCAAD registry.^8^

Similar to previous reports, we found that complete resection of the intimal tear and prosthetic replacement of the ascending aorta (with or without hemiarch) are still the most commonly performed procedures for type A aortic dissections.^3^ ^,^ ^7–9^ In this study, the extension of surgical repair (ascending aorta, hemiarch, total arch replacement) was usually based on the surgical assessment of the aortic pathology and, therefore, surgical repair was aimed at the excision of the dissection entry tear. An entry tear involving the small curvature of the aortic arch was addressed by confectioning an interposition graft with hemiarch. A more extensive arch operation was dictated by the presence of the entry tear near the supra-aortic branches or was based on the surgeon’s ‘judgement call’, after considering patient preoperative conditions, severity of aortic arch involvement and individual surgeon expertise. In recent years, the evolution of subspecialty aortic services and the introduction of new technology with refinements in surgical techniques and cerebral protection have led to extensive surgery for TAAAD that involves aortic arch replacement with possible extensions into the proximal descending aorta along with hybrid, staged procedures.^20^ Extending the first operation for TAAAD to treating the dissected aortas to a greater extent will decrease the risks of progressive aortic dilation and rupture of the residual dissected aorta and offer significant advantage to second stage intravascular treatment options. However, this medium to long term expected benefit from more extensive repairs needs to be weighed against a potential higher mortality and morbidity from the increased complexity of the initial procedure. The present analysis showed that patients who underwent total aortic arch replacement were more likely to experience a fatal event. While total arch replacement has been consistently shown to be safe in elective surgery, controversy still exists about its impact on surgical mortality and morbidity and its long-term benefit in the setting of TAAAD.^21^ Our results confirm the hypothesis that total aortic arch replacement should be undertaken on the basis of the patient preoperative conditions and surgical findings. Compared to other registries, our cohort showed a lower proportion of patients undergoing total arch replacement and this could be attributed to the fact that only few units in the UK currently have a dedicated aortic service delivered by specialist aortic surgeons. As such, most of the operating surgeons may prefer to perform less complex procedures [e.g. interposition graft (± hemiarch)] instead of more technically demanding repairs, whenever possible. Moreover, the current UK policy regarding systematic public reporting of operative outcomes of individual surgeons may generate risk averse behaviours characterized by refraining from performing high-risk procedures potentially associated with worse in-hospital outcomes, such as aortic arch replacement in the setting of TAAAD.^22^

Total root replacement was performed in 24% of patients in our study with no impact on the observed mortality. Despite the anticipated increased technical complexity of aortic root surgery in the setting of TAAAD, other large series have also reported equivalent operative mortality, with a lower incidence of aortic root reoperation.^3^ ^,^ ^7^ ^,^ ^23^ It can therefore be recommended that total aortic root replacement should be considered when indicated (e.g. extensive root destruction, concomitant root aneurysm, bicuspid aortopathy, or history of connective tissue disease).^24^ ^,^ ^25^

Reports from other registries have found that it is the anatomical extension of the tear and not the extension of surgical repair that has an effect on patient outcome.^7^ This, however, could be explained by inherited bias related to the fact that surgeons may decide upon a limited procedure in sick and/or elderly patients vs. a more extensive repair in lower risk and/or younger patients. Moreover, there is no agreement on which features should be considered when we want to account for the extension of the disease (e.g. entry point vs. size vs. extension of the dissection) and this could also contribute to the reported differences in practices and outcomes.

In the present analysis, the cerebral perfusion strategy *per se* was not identified as an independent risk factor for mortality and no difference in postoperative mortality was found in patients with and without cerebral perfusion undergoing hemiarch and arch replacement surgery. Other registries of unselected patients (e.g. STS and GERAADA) have found no association between cerebral perfusion strategies and outcomes.^7^ ^,^ ^19^ Various biases are likely to balance outcome and lead to this observation. For instance, cerebral perfusion could be used in patients requiring more extensive repair and longer circulatory arrest time as in patients undergoing arch replacement. The choice of neuroprotective strategies is mainly tailored on surgeon’s experience and skillset, patient characteristics, and extent of surgical resection. The benefit of cerebral perfusion becomes apparent when the analysis is focused on patients requiring complex procedures, with longer circulatory arrest time (>30 min).^9^ ^,^ ^26–28^

Our analysis confirmed a strong inverse relationship between adjusted mortality and surgeon annual case volume. As the annual number of cases per surgeon has increased, these results suggest that the observed reduction in in-hospital mortality in the most recent years can be partially due to the volume–outcome effect. We found evidence that a minimum of 5 cases per year per surgeon should be sought to improve outcomes. However, our mixed model showed that annual surgeon volume of TAAAD repairs does not fully account for the relationship between individual surgeon and mortality. The surgeon effect remained significant after adjusting for the surgeon annual volume, thus suggesting that other unmeasured factors could have contributed to the observed differences in outcomes. For instance, inadvertent aortic sub-specialization of those surgeons performing a high volume of elective aortic procedures will allow them to develop and improve the skillset and expertise useful in the context of emergency aortic cases. Moreover, this finding highlights the need for surgical training programs to educate and expose the new generations of surgeons to dissection cases due to the importance of surgeon skills in improving outcomes of this dire surgical disease.

The initial correlation between centre volume of operated TAAAD and mortality was no longer significant when the model accounted for the effect related to individual surgeons. This suggests that factors other than centre volume itself may explain the variability observed across different centres (i.e. dedicated aortic service, streamlined perioperative care), including regional differences regarding referral pathways and networking. Highly efficient referral pathways have been proven to influence better outcomes by reducing waiting at peripheral hospitals and transfer times.^29^ The presence of specialized aortic services able to provide 24/7 access for patients presenting with TAAAD represents a relevant factor for the improvement of outcomes in these cases by optimizing the referral pathway and allowing referring centres to have a direct contact with the aortic team.^30^

Finally, we did not find a significant effect of the responsible anaesthetic on outcomes, although it is plausible that the quality of intensive care may still play an important role in determining patient outcomes.

Given the relatively small number of patients with TAAAD referred to surgery every year, most UK cardiac centres are unlikely to build adequate expertise to optimize surgical outcomes. In light of the relevant effect related to individual surgeon and institution on outcomes and the rapid access in most urban centres to surgical teams with focused expertise in the management of aortic dissection, it may no longer be appropriate to give priority to rapid surgical transfer over transfer to an experienced centre. With improvements in diagnostic times and early medical management, access to teams with subspecialty interest in aortic surgery can further reduce operative mortality.

###  

#### Limitations

The reported results are limited by information registered within the database that has been interrogated. Important operative details that might influence patient outcome, such as cannulation sites, cross clamp vs. open distal anastomosis or core temperature, were not recorded. Also, concomitant coronary artery bypass graft surgery included cases where coronary artery involvement was evident at computed tomography, but we were not able to determine whether it was a planned or bail-out intraoperative strategy.

Another limitation is that we do not yet have access to longer-term follow-up data. There is a lack of information about preoperative patient selection, as patients who were not offered surgery were not included in the registry. Finally, the UK aortic score will need external validation in case series from other countries before it can be adopted outside the UK.

## Conclusions

Patient characteristics, intraoperative factors, cardiac centre, and high-volume surgeons are strong determinants of outcomes following TAAAD surgery. These results can help refining clinical decision-making, supporting patient counselling, and can be used by policy makers for service provision improvement.

## Supplementary material

Supplementary material is available at *European Heart Journal* online.

## Supplementary Material

ehab586_Supplementary_Data

## Data Availability

The data underlying this article cannot be shared publicly due to privacy and ethical reasons. The data will be shared on reasonable request to the corresponding author. **Conflict of interest**: none declared.
