# Supplementary material for: Determinants of outcomes following surgery for type A acute aortic dissection: the UK National Adult Cardiac Surgical Audit
Source: Eur Heart J. 2021 Sep 1;43(1):44–52. doi: 10.1093/eurheartj/ehab586 (PMC8720141; doi:10.1093/eurheartj/ehab586)
Supplement: ehab586_Supplementary_Data [file ehab586_supplementary_data.zip › ehab586-Suppl_data/Supplemental Material.docx]

**Supplemental Material**

**Determinants of outcomes following surgery for type A acute aortic dissection: The UK National Adult Cardiac Surgical Audit.**

Benedetto U. et al.

**CONTENT**

**Supplementary Table 1.** Postoperative outcomes in patients undergoing full arch replacement with or without cerebral perfusion.

**Supplementary Table 2.** Postoperative outcomes in patients undergoing hemiarch with or without cerebral perfusion.

**Supplementary Table 3.** Postoperative outcomes in patients undergoing arch replacement with or without adjunct endovascular procedure.

**Supplementary Table 4.** Patients’ characteristics and operative data in survivors and non survivors

**Supplementary Table 5.** Bootstrapping corrected optimism- UK Aortic SCORE model validation

**Supplementary Table 6.** Nested models based on UK Aortic SCORE, annual hospital and surgeon volume and random effects (hospital and surgeon) with relative ANOVA P and incremental χ2

**Supplementary Table 7.** Patients’ characteristics, operative data and outcomes in the overall sample and stratified by annual surgeon volume

**Supplementary Figure 1.** Optimism-corrected bootstrapping validation of the UK Aortic SCORE.

**Supplementary Figure 2.** External validation of the International Registry of Aortic Dissection (IRAD) score in our cohort.

**Supplementary Table 1.** Postoperative outcomes in patients undergoing full arch replacement with or without cerebral perfusion.

|  | Without cerebral perfusion | With cerebral perfusion | p |
| --- | --- | --- | --- |
| n | 15 | 70 |  |
| Operative mortality, n (%) | 3 (20.0) | 14 (20.0) | 1.000 |
| Non-fatal CVA, n (%) | 3 (20.0) | 11 (15.7) | 0.982 |
| Postoperative dialysis, n (%) | 2 (13.3) | 16 (24.2) | 0.566 |
| SWI, n (%) | 0 ( 0.0) | 1 ( 2.2) | 1.000 |
| Re-exploration, n (%) | 2 (14.3) | 13 (24.5) | 0.647 |

**Supplementary Table 2.** Postoperative outcomes in patients undergoing hemiarch with or without cerebral perfusion.

|  | Without cerebral perfusion | With cerebral perfusion | p |
| --- | --- | --- | --- |
| n | 470 | 627 |  |
| Operative mortality, n (%) | 84 (17.9) | 108 (17.2) | 0.842 |
| Non-fatal CVA, n (%) | 50 (10.6) | 70 (11.2) | 0.858 |
| Postoperative dialysis, n (%) | 53 (12.6) | 98 (17.5) | 0.045 |
| SWI, n (%) | 3 ( 0.9) | 7 ( 1.9) | 0.429 |
| Re-exploration, n (%) | 42 (10.0) | 60 (11.8) | 0.460 |

**Supplementary Table 3.** Postoperative outcomes in patients undergoing arch replacement with or without adjunct endovascular procedure.

|  | Without endovascular procedure | With endovascular procedure | p | test |
| --- | --- | --- | --- | --- |
| n | 56 | 68 |  |  |
| Operative mortality, n (%) | 12 (21.4) | 16 (23.5) | 0.950 |  |
| Non-fatal CVA, n (%) | 8 (14.3) | 10 (14.7) | 1.000 |  |
| Postoperative dialysis, n (%) | 10 (20.4) | 14 (21.5) | 1.000 |  |
| SWI, n (%) | 0 ( 0.0) | 1 ( 2.4) | 1.000 |  |
| Re-exploration, n (%) | 7 (14.9) | 11 (20.8) | 0.617 |  |

**Supplementary Table 4.** Patients’ characteristics and operative data in survivors and non survivors

|  | Survivors | Non survivors | p |
| --- | --- | --- | --- |
| n | 3456 | 747 |  |
| Age, median [IQR] | 63.20 [51.70, 72.60] | 67.60 [56.65, 74.90] | <0.001 |
| Age categories, n (%) |  |  | <0.001 |
| 59 or less | 1480 (42.8) | 230 (30.8) |  |
| 60-64 | 412 (11.9) | 94 (12.6) |  |
| 65-69 | 441 (12.8) | 109 (14.6) |  |
| 70-74 | 496 (14.4) | 127 (17.0) |  |
| 75-79 | 424 (12.3) | 113 (15.1) |  |
| 80 or above | 203 ( 5.9) | 74 ( 9.9) |  |
| Female, n (%) | 1140 (33.0) | 259 (34.7) | 0.399 |
| Marfan syndrome, n (%) | 99 ( 2.9) | 13 ( 1.7) | 0.109 |
| Chronic pulmonary disease, n (%) | 271 ( 7.8) | 85 (11.4) | 0.002 |
| Smoking, n (%) |  |  | 0.818 |
| 0. Never smoked | 1782 (51.6) | 390 (52.2) |  |
| 1. Ex-smoker | 1100 (31.8) | 240 (32.1) |  |
| 2. Current smoker | 574 (16.6) | 117 (15.7) |  |
| Any pulse deficit, n (%) | 648 (18.8) | 183 (24.5) | <0.001 |
| Hypertension, n (%) | 2278 (65.9) | 530 (71.0) | 0.009 |
| Diabetes, n (%) |  |  | 0.632 |
| 0. Not Diabetic | 3260 (94.3) | 708 (94.8) |  |
| 1. Diet | 46 ( 1.3) | 6 ( 0.8) |  |
| 2. Oral therapy | 121 ( 3.5) | 28 ( 3.7) |  |
| 3. Insulin | 29 ( 0.8) | 5 ( 0.7) |  |
| CVA, n (%) | 190 ( 5.5) | 57 ( 7.6) | 0.031 |
| LVEF, n (%) |  |  | <0.001 |
| 1. Good (≥50%) | 2834 (82.0) | 515 (68.9) |  |
| 1. Moderate (30-49%) | 546 (15.8) | 175 (23.4) |  |
| 1. Poor (<30%) | 76 ( 2.2) | 57 ( 7.6) |  |
| Chronic kidney disease, n (%) | 116 ( 3.4) | 52 ( 7.0) | <0.001 |
| Previous cardiac surgery, n (%) | 174 ( 5.0) | 80 (10.7) | <0.001 |
| Acute renal failure, n (%) | 84 ( 2.4) | 43 ( 5.8) | <0.001 |
| Preoperative ventilation, n (%) | 114 ( 3.3) | 86 (11.5) | <0.001 |
| Preoperative resuscitation, n (%) | 6 ( 0.2) | 11 ( 1.5) | <0.001 |
| Ongoing chest pain, n (%) | 344 (10.0) | 106 (14.2) | 0.001 |
| MI at presentation, n (%) | 67 ( 1.9) | 57 ( 7.6) | <0.001 |
| Malperfusion*, n (%) | 1142(33.2) | 385 (51.5) | <0.001 |
| TIA in the last 24 hrs, n (%) | 143 ( 4.1) | 33 ( 4.4) | 0.806 |
| Bicuspid aortic valve, n (%) | 85 ( 2.5) | 13 ( 1.7) | 0.295 |
| Aortic regurgitation, n (%) | 998 (28.9) | 211 (28.2) | 0.763 |
| Aortic arch involvement, n (%) | 733 (21.2) | 169 (22.6) | 0.421 |
| Descending aorta involvement, n (%) | 140 ( 4.1) | 48 ( 6.4) | 0.006 |
| Full root replacement, n (%) | 835 (24.2) | 180 (24.1) | 1.000 |
| Aortic root sparing, n (%) | 21 ( 0.6) | 1 ( 0.1) | 0.178 |
| Aortic arch replacement, n (%) | 96 ( 2.8) | 28 ( 3.7) | 0.193 |
| Hybrid frozen elephant trunk, n (%) | 52 ( 1.5) | 16 ( 2.1) | 0.275 |
| Aortic valve replacement, n (%) | 939 (77.3) | 209 (80.1) | 0.367 |
| Type of aortic valve implanted n (%) |  |  | 0.001 |
| 1. Autograft (Ross) | 11 ( 1.2) | 3 ( 1.5) |  |
| 1. Biological | 368 (41.3) | 110 (54.5) |  |
| 1. Homograft | 5 ( 0.6) | 4 ( 2.0) |  |
| 1. Mechanical | 507 (56.9) | 85 (42.1) |  |
| Cerebral perfusion, n (%) | 923 (26.7) | 203 (27.2) | 0.829 |
| Antegrade cerebral perfusion, n (%) | 741 (21.4) | 161 (21.6) | 0.985 |
| CABG, n (%) | 379 (11.0) | 175 (23.4) | <0.001 |
| Eras, n (%) |  |  | 0.214 |
| 2009-2010 | 444 (12.8) | 117 (15.7) |  |
| 2011-2012 | 612 (17.7) | 135 (18.1) |  |
| 2013-2014 | 674 (19.5) | 145 (19.4) |  |
| 2015-2016 | 838 (24.2) | 180 (24.1) |  |
| 2017-2018 | 888 (25.7) | 170 (22.8) |  |
| Hospital annual volume, median [IQR] | 16.00 [11.00, 25.00] | 14.00 [10.00, 21.00] | <0.001 |
| Surgeon annual volume, median [IQR] | 4.00 [2.00, 6.00] | 3.00 [2.00, 5.00] | <0.001 |

* defined as one of the following conditions: cardiac tamponade, low cardiac output or other forms of hypoperfusion

CABG coronary artery bypass grafting; CVA cerebrovascular accidents; LVEF left ventricular ejection fraction; TIA transient ischemic attack.

**Supplementary Table 5.** Bootstrapping corrected optimism- UK Aortic SCORE model validation

|  | index.orig | training | test | optimism | index.corrected | n |
| --- | --- | --- | --- | --- | --- | --- |
| Dxy | 0.3881847 | 0.4080438 | 0.3870253 | 0.0210185 | 0.3671662 | 200 |
| R2 | 0.1212452 | 0.1336799 | 0.1195585 | 0.0141214 | 0.1071239 | 200 |
| Intercept | 0.0000000 | 0.0000000 | -0.0838660 | 0.0838660 | -0.0838660 | 200 |
| Slope | 1.0000000 | 1.0000000 | 0.9397453 | 0.0602547 | 0.9397453 | 200 |
| Emax | 0.0000000 | 0.0000000 | 0.0292597 | 0.0292597 | 0.0292597 | 200 |
| D | 0.0763056 | 0.0844913 | 0.0752008 | 0.0092905 | 0.0670151 | 200 |
| U | -0.0004759 | -0.0004759 | 0.0003235 | -0.0007993 | 0.0003235 | 200 |
| Q | 0.0767814 | 0.0849671 | 0.0748773 | 0.0100898 | 0.0666916 | 200 |
| B | 0.1336802 | 0.1322917 | 0.1340474 | -0.0017557 | 0.1354359 | 200 |
| g | 0.7658889 | 0.8208448 | 0.7691802 | 0.0516645 | 0.7142244 | 200 |
| gp | 0.1115155 | 0.1172773 | 0.1110044 | 0.0062729 | 0.1052426 | 200 |
| AUC | 0.6940923 | 0.7040219 | 0.6935127 | 0.0105093 | 0.6835831 | 200 |

The column “index.orig” indicates the value when calculated using the model fitted to the original and evaluated on the original data. The column “training” gives the mean of the c-statistic across the bootstrap samples. The column “test” gives the mean of the c-statistic when applying the model fitted to the bootstrap datasets but evaluated on the original dataset. The column “optimism” is calculated as the difference of the “training” and “test” columns. Finally, “index.corrected” indicates the optimism corrected estimate of the Somer’s D (Dxy, which is a transformed version of the c-statistic).

Emax: the maximum absolute difference in predicted and calibrated probabilities; D: the discrimination index; U: the unreliability index; Q: overall quality index (Q=D-U); B: Brier or quadratic probability score; g-index: Gini’s mean difference; gp: g-index on the probability scale.

**Supplementary Table 6.** Nested models based on UK Aortic SCORE, annual hospital and surgeon volume and random effects (hospital and surgeon) with relative ANOVA P and incremental χ2

|  | | | **Logit (UK Aortic SCORE)** | | | | | **+surgeon volume** | | | | | **+hospital volume** | | | | + hospital (RE) | | | | | **+ surgeon (RE)** | | | | | **+anesthetist** | | |
| --- | --- | --- | --- | --- | --- | --- | --- | --- | --- | --- | --- | --- | --- | --- | --- | --- | --- | --- | --- | --- | --- | --- | --- | --- | --- | --- | --- | --- | --- |
| *Predictors* | *Odds Ratios* | *CI* | | *p* | | *Odds Ratios* | *CI* | | *p* | | *Odds Ratios* | *CI* | | *p* | | *Odds Ratios* | | *CI* | *p* | | *Odds Ratios* | | *CI* | *p* | | *Odds Ratios* | | *CI* | *p* |
| (Intercept) | 1.00 | 0.83 – 1.20 | | 1.000 | | 1.35 | 1.08 – 1.68 | | **0.008** | | 1.60 | 1.25 – 2.06 | | **<0.001** | | 1.48 | | 1.10 – 2.01 | **0.011** | | 1.56 | | 1.14 – 2.14 | **0.006** | | 1.56 | | 1.14 – 2.14 | **0.006** |
| logit(UK Aortic SCORE) | 2.72 | 2.43 – 3.05 | | **<0.001** | | 2.72 | 2.43 – 3.06 | | **<0.001** | | 2.73 | 2.43 – 3.06 | | **<0.001** | | 2.91 | | 2.58 – 3.28 | **<0.001** | | 3.04 | | 2.68 – 3.45 | **<0.001** | | 3.04 | | 2.68 – 3.45 | **<0.001** |
| Surgeon year volume |  |  | |  | | 0.93 | 0.91 – 0.96 | | **<0.001** | | 0.95 | 0.92 – 0.98 | | **0.003** | | 0.95 | | 0.92 – 0.98 | **0.003** | | 0.95 | | 0.92 – 0.99 | **0.010** | | 0.95 | | 0.92 – 0.99 | **0.010** |
| Hospital year volume |  |  | |  | |  |  | |  | | 0.99 | 0.98 – 0.99 | | **0.003** | | 1.00 | | 0.98 – 1.01 | 0.485 | | 0.99 | | 0.98 – 1.01 | 0.366 | | 0.99 | | 0.98 – 1.01 | 0.366 |
| **Random Effects** | | | | | | | | | | | | | | | | | | | | | | | | | | | | | |
| hospital.sd (Intercept) | | |  | | | | |  | | | | |  | | | | 0.16 | | | | | 0.15 | | | | | 0.15 | | |
| Surgeon sd (Intercept) | | |  | | | | |  | | | | |  | | | |  | | | | | 0.19 | | | | | 0.19 | | |
| Anesthetist .sd (Intercept) | | |  | | | | |  | | | | |  | | | |  | | | | |  | | | | | 0.00 | | |
| ANOVA for nested models P | | |  | | | | | <0.0001 | | | | | 0.003 | | | | <0.0001 | | | | | 0.0015 | | | | | 1 | | |
| ANOVA for nested model χ2 | | |  | | | | | 22 | | | | | 9 | | | | 36 | | | | | 10 | | | | | 0 | | |
| *^1^*OR = Odds Ratio, CI = Confidence Interval | | | | |  | | | | |  | | | | |  | | | | |  | | | | |  | | | | |

**Supplementary Table 7.** Patients’ characteristics, operative data and outcomes in the overall sample and stratified by annual surgeon volume

|  | [1,3] | (3,5] | (5,20] | p |
| --- | --- | --- | --- | --- |
| n | 1916 | 1044 | 1243 |  |
| Surgeon total volume, median [IQR] | 9.00 [5.00, 15.00] | 17.00 [10.00, 24.00] | 32.00 [22.00, 40.00] | <0.001 |
| Surgeon annual volume, median [IQR] | 2.00 [1.00, 3.00] | 4.00 [4.00, 5.00] | 8.00 [6.00, 9.00] | <0.001 |
| Age, median [IQR] | 63.30 [52.10, 72.53] | 63.95 [51.68, 73.12] | 64.90 [54.00, 73.80] | 0.008 |
| Age categories, n (%) |  |  |  | 0.193 |
| 59 or less | 803 (41.9) | 430 (41.2) | 477 (38.4) |  |
| 60-64 | 243 (12.7) | 115 (11.0) | 148 (11.9) |  |
| 65-69 | 250 (13.0) | 146 (14.0) | 154 (12.4) |  |
| 70-74 | 278 (14.5) | 156 (14.9) | 189 (15.2) |  |
| 75-79 | 220 (11.5) | 135 (12.9) | 182 (14.6) |  |
| 80 or above | 122 ( 6.4) | 62 ( 5.9) | 93 ( 7.5) |  |
| Female, n (%) | 623 (32.5) | 325 (31.1) | 451 (36.3) | 0.021 |
| Marfan syndrome, n (%) | 38 ( 2.0) | 32 ( 3.1) | 42 ( 3.4) | 0.038 |
| Chronic pulmonary disease, n (%) | 156 ( 8.1) | 91 ( 8.7) | 109 ( 8.8) | 0.782 |
| Smoking, n (%) |  |  |  | 0.114 |
| 0. Never smoked | 1007 (52.6) | 553 (53.0) | 612 (49.2) |  |
| 1. Ex-smoker | 583 (30.4) | 325 (31.1) | 432 (34.8) |  |
| 2. Current smoker | 326 (17.0) | 166 (15.9) | 199 (16.0) |  |
| Any pulse deficit, n (%) | 380 (19.8) | 196 (18.8) | 255 (20.5) | 0.579 |
| Hypertension, n (%) | 1261 (65.8) | 693 (66.4) | 854 (68.7) | 0.228 |
| Diabetes, n (%) |  |  |  | 0.125 |
| 0. Not Diabetic | 1814 (94.7) | 994 (95.2) | 1160 (93.3) |  |
| 1. Diet | 18 ( 0.9) | 14 ( 1.3) | 20 ( 1.6) |  |
| 2. Oral therapy | 73 ( 3.8) | 26 ( 2.5) | 50 ( 4.0) |  |
| 3. Insulin | 11 ( 0.6) | 10 ( 1.0) | 13 ( 1.0) |  |
| CVA, n (%) | 108 ( 5.6) | 70 ( 6.7) | 69 ( 5.6) | 0.421 |
| LVEF, n (%) |  |  |  | 0.004 |
| 1. Good (≥50%) | 1502 (78.4) | 812 (77.8) | 1035 (83.3) |  |
| 1. Moderate (30-49%) | 344 (18.0) | 196 (18.8) | 181 (14.6) |  |
| 1. Poor (<30%) | 70 ( 3.7) | 36 ( 3.4) | 27 ( 2.2) |  |
| Chronic kidney disease, n (%) | 80 ( 4.2) | 40 ( 3.8) | 48 ( 3.9) | 0.864 |
| Previous cardiac surgery, n (%) | 89 ( 4.6) | 74 ( 7.1) | 91 ( 7.3) | 0.002 |
| Acute renal failure, n (%) | 48 ( 2.5) | 45 ( 4.3) | 34 ( 2.7) | 0.018 |
| Preoperative ventilation, n (%) | 100 ( 5.2) | 53 ( 5.1) | 47 ( 3.8) | 0.153 |
| Preoperative resuscitation, n (%) | 10 ( 0.5) | 5 ( 0.5) | 2 ( 0.2) | 0.268 |
| Ongoing chest pain, n (%) | 215 (11.2) | 103 ( 9.9) | 132 (10.6) | 0.519 |
| MI at presentation, n (%) | 62 ( 3.2) | 40 ( 3.8) | 22 ( 1.8) | 0.009 |
| Malperfusion*, n (%) | 721 (37.6) | 392 (37.5) | 421 (33.9) | 0.07 |
| TIA in the last 24 hrs, n (%) | 82 ( 4.3) | 47 ( 4.5) | 47 ( 3.8) | 0.667 |
| Cardiogenic shock, n (%) | 308 (16.1) | 162 (15.5) | 139 (11.2) | <0.001 |
| Bicuspid aortic valve, n (%) | 39 ( 2.0) | 30 ( 2.9) | 29 ( 2.3) | 0.353 |
| Aortic regurgitation, n (%) | 552 (28.8) | 301 (28.8) | 356 (28.6) | 0.993 |
| Aortic root involvement, n (%) | 554 (28.9) | 273 (26.1) | 346 (27.8) | 0.276 |
| Ascending aorta involvement, n (%) | 1512 (78.9) | 793 (76.0) | 874 (70.3) | <0.001 |
| Aortic arch involvement, n (%) | 336 (17.5) | 226 (21.6) | 340 (27.4) | <0.001 |
| Descending aorta involvement, n (%) | 61 ( 3.2) | 44 ( 4.2) | 83 ( 6.7) | <0.001 |
| Abdominal aorta involvement, n (%) | 27 ( 1.4) | 21 ( 2.0) | 26 ( 2.1) | 0.281 |
| Full root replacement, n (%) | 458 (23.9) | 241 (23.1) | 316 (25.4) | 0.405 |
| Aortic root sparing, n (%) | 9 ( 0.5) | 9 ( 0.9) | 4 ( 0.3) | 0.185 |
| Aortic arch surgery, n (%) | 39 ( 2.0) | 25 ( 2.4) | 60 ( 4.8) | <0.001 |
| Hybrid frozen elephant trunk, n (%) | 12 ( 0.6) | 13 ( 1.2) | 43 ( 3.5) | <0.001 |
| Surgery type, n (%) |  |  |  | <0.001 |
| 1. Aortic arch replacement | 39 ( 2.0) | 25 ( 2.4) | 60 ( 4.8) |  |
| 1. Interposition graft | 1425 (74.4) | 781 (74.8) | 879 (70.7) |  |
| 1. Root replacement | 452 (23.6) | 238 (22.8) | 304 (24.5) |  |
| Aortic valve replacement, n (%) | 525 (77.4) | 285 (76.4) | 338 (79.5) | 0.547 |
| Type of aortic valve implanted, n (%) |  |  |  | <0.001 |
| 1. Autograft (Ross) | 4 ( 0.8) | 8 ( 3.0) | 2 ( 0.6) |  |
| 1. Biological | 188 (37.4) | 137 (50.7) | 153 (47.8) |  |
| 1. Homograft | 3 ( 0.6) | 3 ( 1.1) | 3 ( 0.9) |  |
| 1. Mechanical | 308 (61.2) | 122 (45.2) | 162 (50.6) |  |
| Cerebral perfusion, n (%) | 423 (22.1) | 275 (26.3) | 428 (34.4) | <0.001 |
| Antegrade cerebral perfusion, n (%) | 343 (17.9) | 236 (22.6) | 323 (26.0) | <0.001 |
| CABG, n (%) | 271 (14.1) | 127 (12.2) | 156 (12.6) | 0.231 |
| Eras, n (%) |  |  |  | <0.001 |
| 2009-2010 | 380 (19.8) | 119 (11.4) | 62 ( 5.0) |  |
| 2011-2012 | 411 (21.5) | 181 (17.3) | 155 (12.5) |  |
| 2013-2014 | 347 (18.1) | 199 (19.1) | 273 (22.0) |  |
| 2015-2016 | 369 (19.3) | 309 (29.6) | 340 (27.4) |  |
| 2017-2018 | 409 (21.3) | 236 (22.6) | 413 (33.2) |  |
| Hospital annual volume, median [IQR] | 12.00 [8.00, 17.00] | 17.00 [12.00, 25.00] | 21.00 [16.00, 30.00] | <0.001 |
| Operative mortality, n (%) | 397 (20.7) | 181 (17.3) | 169 (13.6) | <0.001 |
| Non-fatal CVA, n (%) | 185 ( 9.7) | 95 ( 9.1) | 108 ( 8.7) | 0.647 |
| Postoperative dialysis, n (%) | 250 (14.7) | 148 (15.6) | 162 (13.9) | 0.541 |
| SWI (%) | 16 ( 1.5) | 9 ( 1.4) | 8 ( 1.1) | 0.741 |
| Re-exploration, n (%) | 214 (12.7) | 104 (10.9) | 123 (11.5) | 0.332 |

CABG coronary artery bypass grafting; CVA cerebrovascular accidents; LVEF left ventricular ejection fraction; SWI sternal wound infection; TIA transient ischemic attack
