# Supplementary figures and images for: Determinants of outcomes following surgery for type A acute aortic dissection: the UK National Adult Cardiac Surgical Audit
Source: Eur Heart J. 2021 Sep 1;43(1):44–52. doi: 10.1093/eurheartj/ehab586 (PMC8720141; doi:10.1093/eurheartj/ehab586)

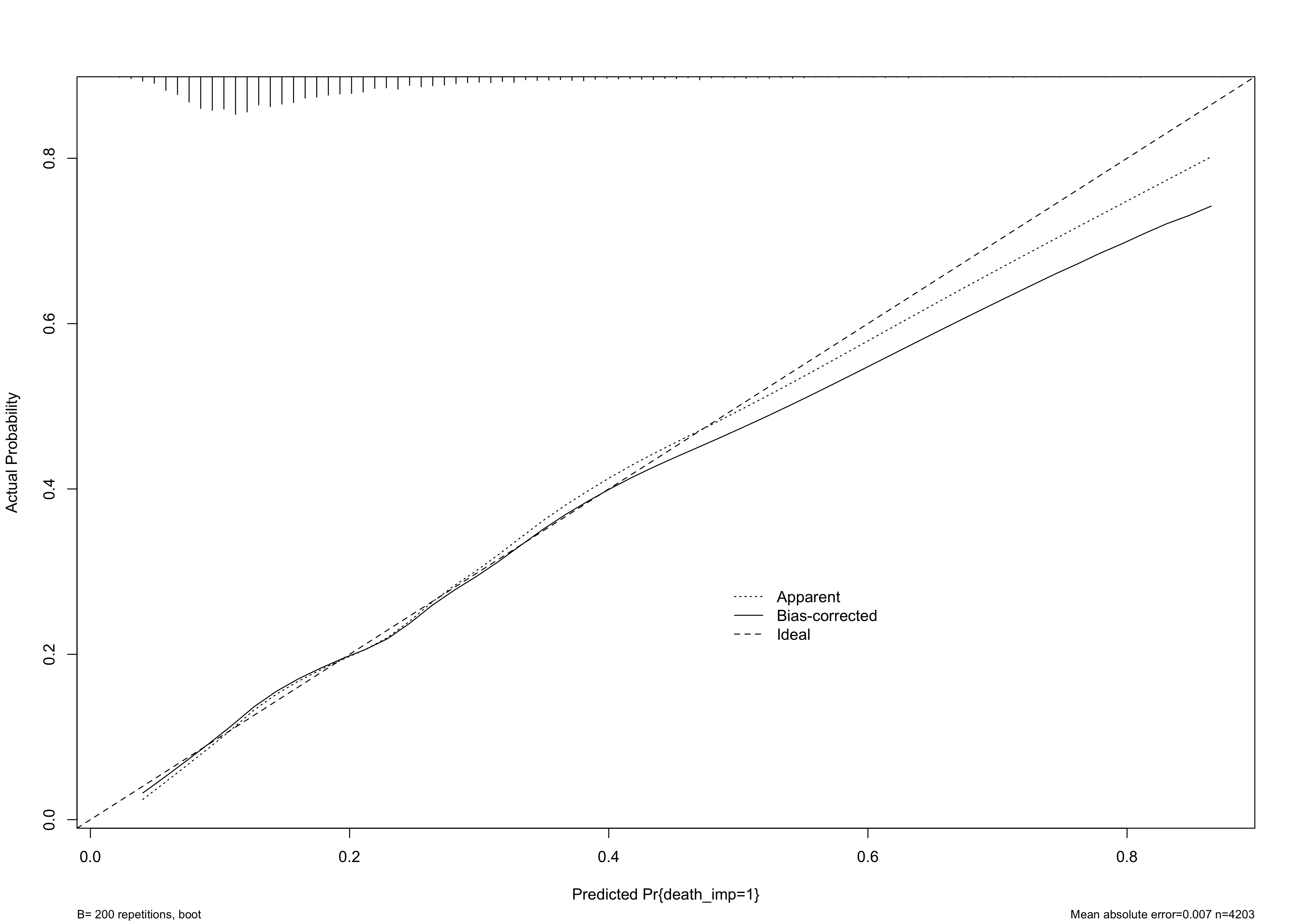

Supplement: ehab586_Supplementary_Data [file ehab586_supplementary_data.zip › ehab586-Suppl_data/Supplementary Figure 1.tiff]

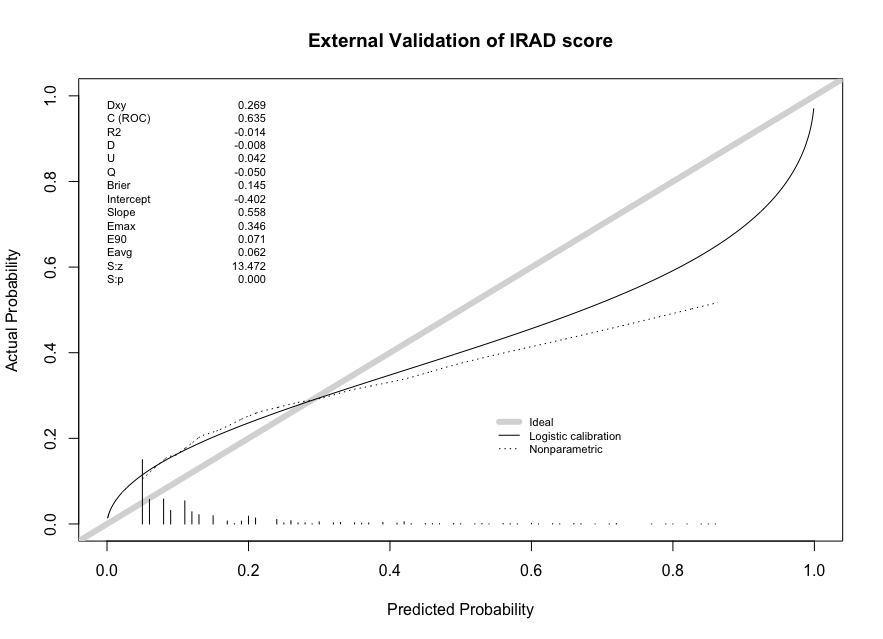

Supplement: ehab586_Supplementary_Data [file ehab586_supplementary_data.zip › ehab586-Suppl_data/Supplementary Figure 2.tiff]
